# Women's Obstetric History and Midtrimester Cervical Length Measurements by 2D/3D and Doppler Ultrasound

**DOI:** 10.1055/s-0040-1713010

**Published:** 2020-06-23

**Authors:** Juliana Valente Codato Marinelli, Antonio Gomes de Amorim Filho, Monica Fairbanks de Barros, Agatha Sacramento Rodrigues, Rossana Pulcineli Vieira Francisco, Mario Henrique Burlacchini de Carvalho

**Affiliations:** 1 Obstetric Clinic Division, Hospital das Clinicas (HCFMUSP), Faculdade de Medicina, Universidade de São Paulo, São Paulo, Brazil; 2 Obstetrics Discipline, Department of Obstetrics and Gynecology, Faculdade de Medicina, Universidade de São Paulo, São Paulo, Brazil

**Keywords:** cervix uteri, cervical length measurement, pregnancy trimester, second, reproductive history, pregnancy, high-risk, colo do útero, medida do comprimento cervical, segundo trimestre da gravidez, história reprodutiva, gravidez de alto risco

## Abstract

**Objective** The aim of the present study was to compare the obstetric history and both two- and tri-dimensional ultrasound parameters according to different cervical lengths.

**Methods** The present cross-sectional study analyzed 248 midtrimester pregnant women according to cervical length and compared the data with the obstetric history and 2D/3D ultrasound parameters. Patients were divided into 3 groups according to cervical length: The Short Cervix group for cervical lengths ≥ 15 mm and < 25 mm ( *n* = 68), the Very Short Cervix group for cervical lengths < 15 mm ( *n* = 18) and the Control group, composed of 162 pregnant women with uterine cervical lengths ≥ 25 mm.

**Results** When analyzing the obstetric history of only non-nulliparous patients, a significant association between the presence of a short cervix in the current pregnancy and at least one previous preterm birth was reported ( *p* = 0.021). Cervical length and volume were positively correlated (Pearson coefficient = 0.587, *p* < 0.0001). The flow index (FI) parameter of cervical vascularization was significantly different between the Control and Very Short Cervix groups. However, after linear regression, in the presence of volume information, we found no association between the groups and FI. Uterine artery Doppler was also not related to cervical shortening.

**Conclusion** The present study showed a significant association between the presence of a short cervix in the current pregnancy and at least one previous preterm birth. None of the vascularization indexes correlate with cervical length as an independent parameter. Uterine artery Doppler findings do not correlate with cervical length.

## Resumo

**Objetivo** O objetivo do presente estudo foi comparar a história obstétrica e os parâmetros bi- e tridimensionais ultrassonográficos de acordo com os diferentes comprimentos cervicais.

**Métodos** O presente estudo transversal analisou 248 gestantes no segundo trimestre de acordo com o comprimento cervical e comparou os dados com a história obstétrica e os parâmetros ultrassonográficos 2D/3D. As pacientes foram divididas em 3 grupos de acordo com o comprimento do colo uterino: grupo Colo Curto para comprimentos cervicais ≥ 15 mm e < 25 mm ( *n* = 68), grupo Colo Muito Curto para comprimentos cervicais < 15 mm ( *n* = 18) e grupo Controle, composto por 162 gestantes com comprimento cervical uterino ≥ 25 mm.

**Resultados** Ao analisar a história obstétrica apenas de pacientes não nulíparas, foi relatada uma associação significativa entre a presença de colo uterino curto na gravidez atual e pelo menos um episódio de parto prematuro anterior ( *p* = 0,021). Comprimento e volume do colo uterino foram correlacionados positivamente (coeficiente de Pearson = 0,587, *p* < 0,0001). O parâmetro índice de fluxo (IF) da vascularização cervical foi significativamente diferente entre os grupos Controle e Colo Muito Curto. Entretanto, após regressão linear, na presença de informações de volume, não encontramos associação entre os grupos e o parâmetro IF. Também não foi encontrada relação entre o Doppler da artéria uterina e o encurtamento cervical.

**Conclusão** O presente estudo mostrou uma associação significativa entre a presença de colo uterino curto na gravidez atual e pelo menos um episódio de parto prematuro anterior. Nenhum dos índices de vascularização se correlaciona com o comprimento cervical como parâmetro independente, assim como o Doppler da artéria uterina também não está relacionado ao comprimento do colo uterino.

## Introduction

The primary mechanical function of the uterine cervix is maintaining pregnancy to term, and the cervix undergoes complex changes during gestation. Understanding the underlying mechanisms of these changes could provide earlier detection of the onset of some complex processes, such as cervical insufficiency and preterm birth. 1

Cervical length determined by transvaginal ultrasound in the second trimester is currently the best predictor of preterm birth. 2 The risks of prematurity increase as the cervix decreases. For a cervical length of ≤ 25 mm, the risk of preterm delivery is between 25 and 30%, but for a cervix < 15 mm the risk is almost 50%. 3 However, the assessment of other cervical ultrasound parameters that are already available and that could even precede cervical shortening remains to be elucidated. Rovas et al 4 studied pregnancies longitudinally and found that 3D cervical vascular indices are stable during pregnancy. There are few data showing that these indexes are different comparing pregnancies in preterm labor and normal development. However, we do not know how these indexes behave in pregnancies of risk for prematurity related to short cervix.

There is evidence that angiogenic factors may also play a key role in cervical ripening.

Uterine artery Doppler sonography analyzes uteroplacental perfusion and may also participate in the remodeling of the cervix. Recent evidence suggests that defective placentation, with failure to transform the myometrial segment of spiral arteries, may be more frequently associated with spontaneous preterm deliveries. 5 6 7

Therefore, the literature is not clear about the contribution of 3D parameters in the evaluation of the cervix during pregnancy and if it is different according to the cervical length. The objective of the present study was to compare the obstetric history and both bi- and tridimensional ultrasound parameters according to different cervical lengths.

## Methods

Cross-sectional study performed in the Fetal Medicine Unit of the Obstetric Clinic of the Hospital das Clínicas of the Faculdade de Medicina da Universidade de São Paulo (FMUSP, in the Portuguese acronym) covering data from May 2014 to January 2018 from the PROPE Project (ClinicalTrials.gov Identifier NCT02511574). The present study is a branch of a main study that compares progesterone and Arabin Pessary for the prevention of preterm delivery in pregnancies with a short cervix. An aleatory subset data of cervical length evaluation before randomization were selected for the present study. The main research project and the present study were approved by the Ethics Committee for the Analysis of Research Projects of the Hospital das Clínicas of the FMUSP (number 1.730.615). Patients (or legal representatives) allocated to Short Cervix and Very Short Cervix groups signed an informed consent form approved by the Ethics Committee for Research Projects Analysis of the hospital. Regarding the Control group, we requested permission to use database information.

Midtrimester pregnant women receiving second trimester anomaly ultrasounds from low- and high-risk clinics underwent a cervical transvaginal evaluation. Patients with cervical lengths < 25 mm were elected for the study and divided into three groups. The groups were divided according to cervical length considering the definition of short cervix when the cervix is < 25 mm and of very short cervix < 15 mm, because the latter group has a 3-fold risk for prematurity. 3 The groups were: The Short Cervix group for cervical lengths ≥ 15 mm and < 25 mm ( *n* = 68), the Very Short Cervix group for cervical lengths < 15 mm ( *n* = 18) and the Control group, composed of 162 pregnant women with uterine cervical lengths ≥ 25 mm. The number of patients in the Control group corresponded to the total number of pregnant women with ≥ 25 mm cervical length assessed during the study period and about whom we had proper information to compare with that of the other two groups.

The inclusion criteria were singleton living fetus pregnancy without malformations, between 20 and 23 weeks and 6 days of gestation established by ultrasound performed in the 1 ^st^ trimester or 2 ultrasound screenings between 16 and 20 weeks and no history of cervical insufficiency/surgery or preterm rupture of membranes (PROM).

Uterine cervical length was assessed using the transvaginal ultrasound technique with the patient placed in the dorsal lithotomy position with an empty bladder. An ultrasound probe was introduced into the vagina, and care was taken to avoid undue pressure to the cervix. After a satisfactory sagittal image was taken, the transducer was slightly withdrawn until the image became blurred and returned to a perfect image showing the internal os, the cervical canal and the external os. The measurement was placed from the outer to the inner cervical os, including only the segment of the cervical canal that was bordered by the endocervical mucosa. The image occupied ∼ 75% of the screen as described by To et al. 8 For 3D assessment of cervical volume and vascularization indices, we performed real-time screening with virtual organ computer-aided analysis (VOCAL) volumetric assessment. All cervical measurements were performed on multiplanar images, and the contour mode of VOCAL was set to manual, rotation steps at an angle of 30°, that is, six contours of the cervix were drawn manually using the roller ball cursor of the machine. Care was taken not to include the lower uterine segment or the vaginal wall. After all contours were drawn, the volume and power Doppler flow indexes of the cervix were computed automatically. 4 The following blood flow indices were obtained: vascularization index (VI), flow index (FI) and vascularization flow index (VFI). To assess the uterine artery, we used Doppler as recommended by the practical guidelines of International Society of Ultrasound in Obstetrics and Gynecology (ISUOG). 9 In each uterine artery, we assessed the resistance index (RI), pulsatility index (PI) and systolic/diastolic ratio (S/D). All examinations were performed by a single medical sonographer.

The tests were performed using Voluson E8 Expert TM equipment (GE Healthcare, Zipf Austria) with a 5 to 9 MHz transvaginal transducer with a 146° field of view (GE Healthcare, Zipf, Austria). The following identical preinstalled settings were used for all patients: a frequency between 3 and 9 MHz, a pulse repetition frequency of 0.6 kHz, a gain of 5.0, and a low wall motion filter of 1. All information was recorded in a computer database.

The patients were assessed according to their demographic characteristics, obstetric history and ultrasound parameters.

Quantitative variables are summarized through the mean, median, standard deviation (SD), minimum and maximum values. Qualitative variables are presented as the absolute frequency ( *n* ) and percentage (%).

A nonparametric Kruskal-Wallis test was used to compare the quantitative variables in the three groups. To make paired comparisons (multiple comparisons) after the Kruskal-Wallis test (in case of significant results), we considered the Dunn test. The Pearson chi-squared test or the Fisher exact test were used to correlate qualitative variables whenever appropriate. The analysis of linear correlation between two quantitative variables was performed by using the Pearson linear correlation coefficient.

To analyze the consistency of possible significant results of groups in ultrasound parameters, linear regression models were adjusted considering the control variables to evaluate whether the group would remain significant in the presence of any possible confounding variables.

The interclass coefficient was calculated as the intraobserver reproducibility comparing the difference between analyses in 2 different 3D acquisitions.

A 5% significance level was chosen, and the statistical analysis was conducted with IBM SPSS for Windows, Version 20.0 (IBM Corp, Armonk, NY, USA).

The present study was submitted to the Ethics Committee of the Department of Obstetrics and Gynecology of the FMUSP and the Ethics Committee for Research Project Analysis (CAPPesq, in the Portuguese acronym). Participating pregnant women (or legal representatives) signed the Informed Consent Form. To use data from the Control Group, an addendum to the research project was made and consent to use the database was requested.

## Results

The final analysis was performed with 68 (27.42%) pregnant women in the Short Cervix group, 18 (7.26%) in the Very Short Cervix group and 162 (65.32%) in the Control group.

The median cervical length was 34.60 mm (variation, 26.20–54.70) for the Control group, 21.00 mm (variation, 15.10–24.50) for the Short Cervix group, and 10.45 mm (variation, 6.30–14.00) for the Very Short Cervix group.

The groups differed in maternal age, ethnicity, and gestational age at inclusion ( Table 1 ).

**Table 1:** Demographic characteristics of pregnant women according to the transvaginal assessment of uterine cervical length between 20 and 23 weeks and 6 days

Demographic characteristics			Control	Short Cervix	Very Short Cervix	*p* - *value*
			(≥ 25 mm)	(≥ 15 mm and < 25 mm)	(< 15 mm)	
			n = 162	n = 68	n = 18	
Maternal age (years)		Median (minimum–maximum)	31 (14–47)	29.50 (13–41)	30.50 (15–40)	0.025 *
Weight (kg)		Median (minimum–maximum)	69.30 (43–130.20)	66 (49–103)	68.20 (56–107.80)	0.464 *
Height (cm)		Median (minimum–maximum)	161 (145–178)	162.50 (152–181)	164 (150–170)	0.114 *
BMI (kg/m ^2^ )		Median (minimum–maximum)	26.84 (17.55–48.93)	25.97 (18–39.13)	25.53 (20.57–39.12)	0.199 *
Race	Caucasian	n (%)	76 (46.9%)	37 (54.4%)	9 (50%)	0.037**
	Mixed		62 (38.3%)	23 (33.8%)	2 (11.1%)	
	Black		24 (14.8%)	8 (11.8%)	7 (38.9%)	
Smoking		n (%)	5 (3.1%)	3 (4.4%)	0 (0%)	0.836 **
Gestational age at inclusion (weeks)		Median (minimum–maximum))	22.14 (20–23.86)	22.71 (20.29–23.86)	22.08 (20.14–23.86)	0.042 *

* Kruskal-Wallis Test.

** Fisher Exact Test.

According to previous obstetric history (presence of at least one previous episode of pregnancy, delivery, abortion, curettage and/or bleeding), there were no significant differences among the three studied groups ( Table 2 ).

**Table 2:** Obstetric history of pregnant women according to the transvaginal assessment of uterine cervical length between 20 and 23 weeks and 6 days

Obstetric history	GROUP			*p* - *value* *
	Control	Short Cervix	Very Short Cervix	
	(≥ 25 mm)	(≥ 15 mm and < 25 mm)	(< 15 mm)	
	n = 162	n = 68	n = 18	
First pregnancy	59 (36.4%)	29 (42.6%)	8 (44.4%)	0.593
Previous delivery **	90 (55.60%)	29 (42.6%)	8 (44.4%)	0.169
Abortion **	45 (27.8%)	24 (35.3%)	6 (33.3%)	0.508
Curettage **	27 (16.7%)	18 (26.5%)	5 (27.8%)	0.178
Bleeding ***	45 (27.8%)	16 (23.5%)	6 (33.3%)	0.661

* Pearson chi-square test.

** At least one previous episode.

*** At least 1 episode in the current pregnancy.

When analyzing the obstetric history of only non-nulliparous patients, we observed a significant association between the presence of a short cervix in the current pregnancy and at least one previous preterm birth. In the Control group, only 22.2% of the non-nulliparous women had had previous preterm deliveries, whereas in the Short and Very Short Cervix groups, the rates were 48.3% and 37.5%, respectively ( *p* = 0.021).

Regarding the sonographic parameters, we observed a moderate positive linear correlation between the volume and length of the cervix (Pearson coefficient = 0.587, *p* < 0.0001). The correlation between these two measures may be presented by the square equation in which expected volume = 12.214 + 0.968* length, that is, the expected volume of a case with null length is 12.214 cm ^3^ . For each increase of one cervical length unit (mm), an increase of 0.968 volume units (cm ^3^ ) would be expected.

The Control, Short Cervix, and Very Short Cervix groups showed differences in the median volume (43. 8 versus 30.87 versus 19.57, respectively) (p = < 0.001) and median FI parameter of cervical vascularization (38.92 versus 39.32 versus 35.16, respectively) ( *p* = 0.027), and the difference between the Control and Very Short Cervix groups was statistically significant. However, after linear regression, in the presence of volume information, we found no association between the groups and FI. There was no statistical correlation between the groups and the uterine artery Doppler results ( Table 3 ).

**Table 3:** Ultrasound parameters according to the transvaginal assessment of uterine cervical length between 20 and 23 weeks and 6 days

**Parameters**	Group **	**Median**	*p* -value *
		**min–max**	
Volume (cm3)	Control	43. 8 (23.10–100.87)	< 0.001
	Short Cervix	30.87 (7.58–69.04)	
	Very Short Cervix	19.57 (5.42–47.23)	
Vascularization Index (VI)	Control	4.87 (0.51–19.87)	0.656
	Short Cervix	4.10 (0.43–24.23)	
	Very Short Cervix	5.89 (0.41–11.67)	
Vascularization Index (FI)	Control	38.92 (29.02–69.39)	0.027
	Short Cervix	39.32 (28.45–52.44)	
	Very Short Cervix	35.16 (28.71–49.24)	
Vascularization Index (VFI)	Control	2.51 (0.15–7.51)	0.457
	Short Cervix	2.02 (0.13–21.13)	
	Very Short Cervix	2.17 (0.12–5.31)	
Right Uterine Artery (RI)	Control	0.59 (0.38–0.82)	0.075
	Short Cervix	0.59 (0.38–0.90)	
	Very Short Cervix	0.68 (0.41–1.46)	
Right Uterine Artery (S/D)	Control	2.46 (1.61–5.60)	0.197
	Short Cervix	2.53 (1.61–9.85)	
	Very Short Cervix	3.09 (1.71–6.82)	
Right Uterine Artery (PI)	Control	0.97 (0.43–2.39)	0.575
	Short Cervix	0.98 (0.48–3.26)	
	Very Short Cervix	1.05 (0.47–2.77)	
Left Uterine Artery (RI)	Control	0.62 (0.39–0.93)	0.356
	Short Cervix	0.63 (0.41–0.90)	
	Very Short Cervix	0.59 (0.43–0.83)	
Left Uterine Artery (S/D)	Control	2.61 (1.64–4.99)	0.247
	Short Cervix	2.63 (1.69–9.79)	
	Very Short Cervix	2.42 (1.74–5.88)	
Left Uterine Artery (PI)	Control	1.01 (0.51–2.14)	0.194
	Short Cervix	1.04 (0.55–3.94)	
	Very Short Cervix	0.97 (0.61–2.45)	

* Kruskal-Wallis Test.

** Group: Control (n = 162), Short Cervix (n = 68) Very Short Cervix (n = 18).

Abreviations: FI, flow index; VFI, vascularization flow index; RI, resistance index; S/D: systolic/diastolic ratio; PI, pulsatility index.

After adjusting the linear regression model to the FI index with covariables maternal age, race, gestational age at inclusion, history of at least one previous preterm delivery and volume, in addition to group, we noticed that only volume was significant (coefficient 0.14; standard error 0.027; *p* < 0.001), which means that, in the presence of volume information, there was no association between the groups and FI. In the Control and Short Cervix groups, cervical primigravidae had a shorter median volume compared no primigravidae women (Control group volume: 41.0 × 45.2cm ^3^ , *p* = 0.003; Short Cervix group volume: 26.6 × 33.6cm ^3^ , *p* = 0.033).

The intraclass coefficients for the intraobserver repeatability were 0.957 (95% confidence interval (CI): 0.893–0.983) for volume, 0.848 (95%CI: 0.622–0.939) for VI, 0.876 (95%CI: 0.693–0.951) for FI and 0.805 (95%CI: 0.515–0.922) for IVF.

## Discussion

Evaluation of cervical length in the second trimester of pregnancy identifies pregnant women with a high risk for preterm delivery; however, fewer than 20% of pregnant women with a short cervix will have preterm deliveries. 10 Thus, the identification of other findings related to cervical shortening may contribute to early diagnosis and improvement in accurately identifying short-cervix pregnant women who effectively have an increased risk of prematurity and are eligible for treatment.

In our study, transversally selected pregnancies that were screened for prematurity in the 2 ^nd^ trimester by cervical length using a cutoff of < 25 mm were selected. Cervical length < 25 mm is defined as a short cervix and has a 3-fold higher risk for preterm delivery compared with cervix length ≥ 25 mm. 3 Obstetric history and 3D/4D ultrasound parameters were compared between the groups with short and normal lengths, dividing the short cervix groups into Very Short Cervix when the cervix was < 15 mm and Short Cervix when the measurement was between 15 and 24.9 mm. The option to create these two subgroups of short cervices was related to the fact that the shorter the cervix, the higher is the risk for prematurity, and the group with lengths < 15 mm represented the group with higher risk. In the Very Short Cervix group, the median cervical length observed was 10.45 mm, compared with 21 mm in the Short Cervix group. These two groups have completely different risks and therefore should be analyzed separately. The median cervical length in the control group, 34.60 mm, was similar to that reported in other studies. 3 11 12 13 There was a significant difference concerning ethnicity among the three groups, with a higher proportion of afrodescendant women in the Very Short Cervix group. The literature shows an increased incidence of preterm deliveries in afrodescendant women. However, two large studies have reported that when social and demographic factors are considered, ethnic origin is not significant. 14 15 A previous study performed in the Brazilian population has not shown differences in cervical length among afrodescendants and Caucasian women. 16 The present study could not clarify whether the shortening of the cervix in the 2 ^nd^ trimester of pregnancy in afrodescendants was related to race itself or to social factors relevant to the majority of this race worldwide. Nevertheless, efforts should be made to elucidate this condition because if a short cervix is truly found more commonly in afrodescendants independently of the cause, this subgroup of pregnant women could have their cervical lengths monitored.

Concerning maternal age, the literature reports a greater incidence of short cervix and increased risk for prematurity in adolescents. It is suggested that this fact is due to social and behavioral factors and not intrinsic biological determinants of age. 16 17 In our study, the maternal age was statistically younger in the Short Cervix group, but in the Control and Very Short Cervix groups, there were no significant differences. This finding may be explained by the considerably smaller number of pregnant women in the Very Short Cervix group; however, the finding would also be possibly related to the increased risk of prematurity in young pregnant women reported in other studies.

We also found differences between the groups concerning gestational age at inclusion, but this factor was considered clinically irrelevant as it was assessed during a 1-week interval in all women during the screening period and may reflect only the dates the patients were referred for screening.

Even though the literature shows a higher incidence of short cervix and preterm deliveries in smokers, we did not notice differences among the study groups. This may have been due to the low incidence of smokers in the group or the omission of such information by the subjects. 16 18 19 20

Concerning obstetric history, we found an association between pregnant women who had had at least one previous preterm delivery and short cervix in the current pregnancy. This finding confirms that an important risk factor of preterm delivery is a previous history of prematurity. It could be hypothesized that a woman with high risk due to a previous history of prematurity has an increased likelihood to be found with a short cervix in the next pregnancy, and therefore, two strong risk factors could potentialize recurrence. 21 22 23 24 25

The use of 3D techniques is still recent, and there are only few data in the literature describing their use and benefits in the field. Thus, studies on cervical volume in normal pregnancy are still insufficient. In our study, the assessment of transvaginal cervical volumes by the 3D VOCAL technique showed a positive correlation between cervical length and volume, which is expected because a short cervix has a lower volume and the opposite is also true. Therefore, the use of VOCAL requires 3D software and machines that increase costs in medical assistance and probably do not contribute to better prediction, as these parameters are dependent. Our findings are in agreement with other studies that have attempted to increase accuracy in predicting preterm deliveries using the 3D technique to assess the uterine cervix. 26 27 These studies did not show any benefit of using 3D compared with 2D techniques in the field. 27 28 29

Dilek et al 28 observed significantly lower values of length and cervical volume in pregnant women who had spontaneous preterm deliveries than in pregnant women with term deliveries. However, the measurement of cervical volume, calculated by the 2D technique in the referred study, did not add benefits to assessing cervical length for predicting preterm deliveries. Strauss et al 30 observed, in multiple pregnancies, a significant correlation between the mean cervical length assessed by 2D analysis and the mean cervical volume, both assessed abdominally.

Concerning 3D vascular indexes, we observed correlations of different cervical lengths only for FI, with lower FI indexes in the Very Short Cervix group than in the Control group, but the difference was no longer significant after the linear regression analysis. This finding is probably in agreement with other studies that showed that FI is not a perfusion indicator and cannot provide information about the blood volume pumped into vessels during a specific period. In fact, the literature reports that the actual meaning of FI is not clear and that it is less predictable than VI or VFI. 31

It is inferred that the cervix should increase vascularization and flow in preparation for labor; however, studies have not agreed in their data. Rovas et al 4 demonstrated the constant distribution of vascular indices throughout normal pregnancy, and the values did not increase as the pregnancy progressed. De Diego et al observed an increase in VI and VFI in pregnant women with a history of treated preterm labor compared with asymptomatic women with the same cervical length. The FI was higher in asymptomatic women. 32

Studies correlating uterine artery Doppler flow and preterm delivery have shown contradictory results. When assessed in the 1 ^st^ and 2 ^nd^ trimester, uterine artery Doppler flow did not present a significant correlation with spontaneous preterm delivery compared with maternal demographic characteristics and previous obstetric history. 33 34

In our study, there was no significant correlation between uterine artery Doppler and cervical length; therefore, there is probably no association with preterm delivery due to a short cervix.

The present study was performed with a homogenous sample in a single center and showed the behavior of different ultrasound parameters according to uterine cervical length. Although the small sample was a limitation, the present study shows that the parameters analyzed could not be useful for the explanation of cervical shortening. The prediction of preterm delivery was not the objective of the present analysis. The scarce data available in the literature for comparison with our findings is also another difficulty. In the present study, only 3D cervical volume was related to cervical length, which is not new in the literature and is fully expected.

## Conclusion

There is a significant association between the presence of a short cervix in the current pregnancy and at least one previous preterm birth. Cervical length and volume are positively correlated. None of the vascularization indices correlate with cervical length as an independent parameter. Uterine artery Doppler findings do not correlate with cervical length.
